# Double-Bond Character of Phosphates in Solid and Liquid Phases Probed by Oxygen K‑Edge X‑ray Absorption Spectroscopy

**DOI:** 10.1021/acs.jpclett.6c00737

**Published:** 2026-06-01

**Authors:** Masanari Nagasaka, Fumitoshi Kumaki, Jun-ichi Adachi

**Affiliations:** † 88301 Institute for Molecular Science, Okazaki 444-8585, Japan; ‡ Molecular Science Program, Graduate Institute for Advanced Studies, SOKENDAI, Okazaki 444-8585, Japan; § Institute of Materials Structure Science, High Energy Accelerator Research Organization, Tsukuba, Ibaraki 305-0801, Japan; ∥ Department of Chemistry, Keio University, Yokohama 223-8522, Japan; ⊥ Materials Structure Science Program, Graduate Institute for Advanced Studies, SOKENDAI, Tsukuba, Ibaraki 305-0801, Japan

## Abstract

Double-bond character of phosphate groups has important roles for biological reactions and was investigated using the P=O π* peaks in the oxygen K-edge X-ray absorption spectroscopy (XAS). The O K-edge XAS spectra of phosphates in the solid phase found that the double-bond character of phosphates is enhanced as the negative charge increases, where the weak P=O π* peak was observed in NaH_2_PO_4_, but distinct P=O π* peaks were obtained in Na_2_HPO_4_ and Na_3_PO_4_. Conversely, the P=O π* peaks were not observed in the XAS spectra of any phosphates in the liquid phase, indicating a substantial reduction in the double-bond character of the phosphate groups owing to the interaction of Na^+^ ions and the formation of hydration structures. The XAS spectra of diphosphates validated that change in the double-bond character of the phosphate groups is a general phenomenon also observed in high-energy phosphate compounds, which is closely related to the reactivity of adenosine triphosphate hydrolysis.

## Graphical abstract

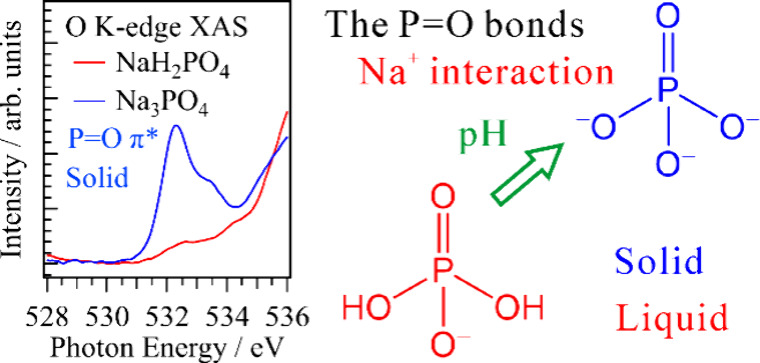

Adenosine triphosphate (ATP) is a high-energy phosphoric compound essential for energy production and transport in most living cells.^1–3^ ATP hydrolysis occurs under moderate physiological conditions, during which the high-energy phosphate bond in ATP is broken by the reaction of water to form adenosine diphosphate and phosphoric acid.^4,5^ The reactivity of ATP hydrolysis is known to increase at pH values exceeding 8.^6^ This enhanced reactivity is associated with changes in the double-bond character of the phosphate groups, which are converted to anions at high pH values. The mechanism of ATP hydrolysis has been extensively studied via several theoretical approaches.^7–9^ Quantum mechanical/molecular mechanical simulations have indicated that the hydration structure of ATP suppresses ATP hydrolysis under moderate physiological conditions despite the P–O bond dissociation associated with high-energy emission.^9^ Understanding the correlation between the double-bound character of phosphate groups and the reactivity of biochemical processes is crucial for elucidating the mechanisms underlying various biological phenomena. Molecular structures of different phosphates have been studied from vibrational structures using infrared spectroscopy^10^ and Raman spectroscopy.^11,12^ The hydration structures of phosphate ions in aqueous solutions have been determined using neutron scattering.^13^ The ^31^P nuclear magnetic resonance determined molecular structures of phosphates,^14,15^ where different phosphate ions were distinguished from the difference of ionic strength. As described above, the molecular structures of phosphates have been studied using several experimental methods, but the electronic structures of phosphates such as P=O orbitals should be investigated for discussing double-bond character of phosphates.

X-ray absorption spectroscopy (XAS) enables element-selective analyses of electronic structures of materials.^16^ Recently, the electronic structures of phosphates in solid and liquid phases have been investigated employing phosphorus K-edge XAS in the hard X-ray region.^17–21^ The P K-edge X-ray emission spectroscopy has also been applied to biomolecules containing phosphate groups such as ATP in aqueous solution.^22^ Spectral differences among different phosphates such as H_2_PO_4_^–^, HPO_4_^2–^, and PO_4_^3–^ are relatively small in P K-edge XAS spectra.^18,20^ Similarly, the spectral changes between the solid and liquid phases of phosphates are minimal in the P K-edge XAS spectra.^18^

The electronic structures of the P=O orbitals in phosphate groups can be investigated using the O K-edge XAS in the soft X-ray region. Because soft X-ray regions below 1 keV includes K-edges of C, N, and O, the soft X-ray XAS method is effective for investigating the electronic structures of water and organic molecules and has been recently applied to liquids and solutions.^23–25^ The hydrogen bond interactions of liquid water have been probed using the pre-edge peaks, which reflect the transition of O 1s electrons to the 4a_1_ unoccupied orbitals, in O K-edge XAS.^26,27^ The double-bond character of phosphate groups can be investigated on the P=O π* peaks, resulting from the transition of O 1s electrons to the P=O π* orbitals.^28^ For comparison, the C=O π* peaks in acetic acid^29^ and the S=O π* peaks in dimethyl sulfoxide (DMSO)^30–32^ appear at energies below the lowest peak corresponding to liquid water,^26,27^ enabling these transitions in liquid phases by separating the contribution of solvent water. Although the sulfinyl group in DMSO has been described as exhibiting both double-bonded (S=O) and zwitterionic (S^+^–O^–^) forms,^33,34^ the strong S=O π* peaks in O K-edge XAS of DMSO validates its significant double-bond character.^30–32^ Similarly, the C=O π* peaks have been observed in polymers^35^ and nucleotides^36,37^ employing O K-edge XAS. Conversely, the P=O π* peak is not resolved in the O K-edge XAS spectrum of solid NaH_2_PO4.^36^ Although aqueous ATP solution has been investigated employing C and N K-edge XAS,^38,39^ it has not been applied to the O K-edge XAS, despite the crucial role of the phosphate group in ATP hydrolysis.

In this study, the double-bond character of phosphates in the solid phase was investigated based on the P=O π* peaks observed in O K-edge XAS, which identified variations in the double-bond character at different phosphates using the combination of inner-shell calculations. The influence of double-bond character on the formation of the hydration structures of phosphates was investigated employing O K-edge XAS in the liquid phase. The relationship between the double-bond character of the phosphate groups and the reactivity of ATP hydrolysis was explored by determining whether changes in the double-bond character of phosphate groups represent a general phenomenon applicable to high-energy phosphate compounds, based on O K-edge XAS of solid diphosphates.

Figure 1(a) shows the O K-edge XAS spectra of phosphates in the solid phase with high purity (>99.0%). The XAS spectra of solid samples were obtained in total electron yield by measuring sample drain currents following soft X-ray absorption, which were conducted at the soft X-ray beamline BL3U of the UVSOR-III Synchrotron.^40^ The details of the sample preparation and the experimental setup were described in Sec. S1 of the Supporting Information. The XAS spectra of solid phosphates consist of the P=O π* peaks that reflect the transition of the O 1s electrons to the P=O π* unoccupied orbitals in the energy region between 530 and 536 eV. The broad peaks above 536 eV would be assigned as the transitions of the O 1s electrons to the P–O σ* orbitals and Rydberg orbitals. The O K-edge XAS spectrum of solid NaH_2_PO_4_ shows two weak P=O π* peaks, whose energetic positions are 532.6 and 534.3 eV. The peak at 534.3 eV would be contributed from the P–OH bonds. These features were consistent with previous experiments.^36^ The O K-edge XAS spectrum of solid Na_2_HPO_4_ exhibits a broad profile around the energy region of the P=O π* peaks, where two main peaks were observed at 532.5 and 533.9 eV. The increase of the P=O π* peak indicates the development of double-bond character in the phosphate groups. Solid Na_3_PO_4_ exhibits the prominent P=O π* peak at 532.3 eV and the shoulder peak at 533.4 eV in the O K-edge XAS spectrum. Note that the P=O π* peak intensity of solid Na_3_PO_4_·12H_2_O was decreased owing to the interactions of water molecules, as described in Sec. S2 of the Supporting Information. These results indicate that the double-bond character of the phosphate groups is enhanced as the negative charges increase, following the order of H_2_PO_4_^–^, HPO_4_^2–^, and PO_4_^3–^.

**Figure 1:**
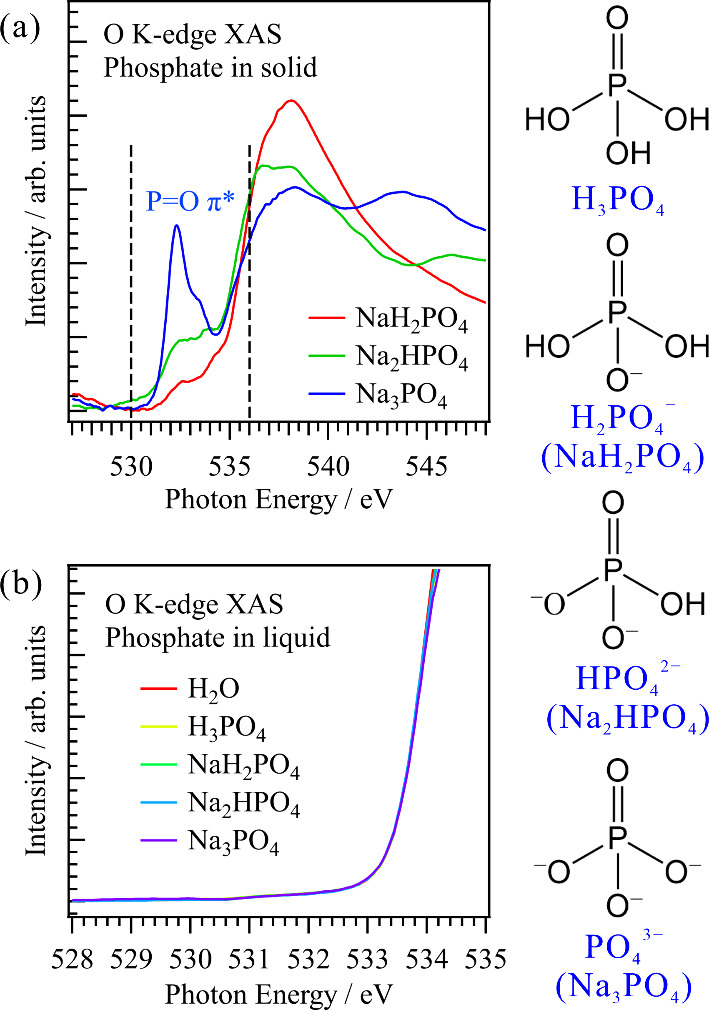
(a) O K-edge XAS spectra of phosphates in solid phases. The energy regions of the P=O π* peaks exist between two dashed lines. (b) O K-edge XAS spectra of phosphates in liquid phases, which were prepared via pH titration. The inset shows the molecular structures of phosphates.

Figure 1(b) shows the O K-edge XAS spectra of phosphates in liquid phases at 25 °C. The XAS spectra of liquid samples were measured utilizing a transmission-type liquid cell comprising two 100 nm-thick Si_3_N_4_ membranes,^24,25^ which were conducted at the soft X-ray beamline BL-7A of KEK-PF.^41^ The phosphate ions were prepared based on the pH titration shown in Sec. S3 of the Supporting Information. The solutions of H_3_PO_4_ with pH of 1.53, H_2_PO_4_^–^ with pH of 5.38, HPO_4_^2–^ with pH of 9.99, and PO_4_^3–^ with pH of 12.22 were prepared by neutralizing 200 mM phosphoric acid with 1 M NaOH. The strong absorbance at approximately 535 eV corresponds to the pre-edge peak of liquid water.^26,27^ The thickness of the liquid layer in the liquid cell is controllable from 20 nm to 40 μm to optimize soft X-ray absorption of liquid samples, where thick layers are prepared for dilute solutions and thin layers are used for condensed solutions.^24,25^ The thickness of the liquid water layer increased above 1 μm to observe the P=O π* peaks of phosphate ions clearly. Therefore, soft X-rays above 535 eV were completely absorbed by liquid water, resulting in no spectral profiles above 535 eV shown in Figure 1(b). In contrast to solid Na_3_PO_4_, no P=O π* peaks were observed in the XAS spectrum of the PO_4_^3–^ solution. Likewise, the P=O π* peaks were not observed for other phosphates in the liquid phase. Because the C=O π* peak in O K-edge XAS of aqueous organic solution is detectable above the concentration of 100 mM,^35^ the present concentration of the PO_4_^3–^ solution (133 mM) exceeds the detection threshold even if PO_4_^3–^ ion has one P=O bond. Note that the P=O π* peaks were also not observed in the condensed 1 M Na_3_PO_4_ aqueous solution, as shown in Sec. S4 of the Supporting Information. These results indicate that the double-bond character of phosphate groups is substantially decreased by the formation of hydration structures in aqueous solutions.

Figure 2(a) shows the O K-edge inner-shell spectra of isolated phosphate molecules, which were obtained by the calculations of the ground and core-excited states through the Hartree–Fock method, ΔSCF (self-consistent field) using the program package GSCF3.^42,43^ The inner-shell spectra were obtained by the summation of four spectra that excited the O 1s electrons of different oxygen atoms in the phosphate groups to the LUMO orbitals. The present calculations focus on the LUMO orbitals because the ΔSCF method has the difficulty for the higher inner-shell excited state with quantitative accuracy. The ΔSCF method is advantageous to reflect the interactions of phosphates with solvent water molecules compared to other inner-shell calculation methods.^44^ Photon energies were referenced to the first peak of PO_4_^3–^ at 532.3 eV, as observed in the XAS spectrum of solid Na_3_PO_4_ shown in Figure 1(a). Figure 2(a) also shows the energy regions of the P=O π* peaks in the experimentally obtained XAS spectra of solid phosphates shown in Figure 1(a) for comparison. The details of the theoretical methods were described in Sec. S1 of the Supporting Information. The calculated charge distributions and the bond character of LUMO orbitals in isolated phosphate molecules were described in Secs. S5 and S6 of the Supporting Information, respectively. The H_3_PO_4_ molecule comprises two types of oxygen atoms, P=O and P–OH, with the energies corresponding to the lowest peaks calculated as 531.805 and 533.323 eV, respectively. The P–OH peak is more intense than that of the P=O peak and lies closer to the absorption edge of liquid water. The P=O peak in the H_3_PO_4_ molecule shows double-bond character, and the P–OH peak shows single-bond character. The energetic position of the P–OH peak is close to the LUMO+1 peak of the P=O group. Note that H_3_PO_4_ molecules only exist in aqueous solutions, and the P=O peak is not observed due to the interactions of the H_3_PO_4_ molecules with solvent water molecules. In the H_2_PO_4_^–^ ion, the P=O peak shifts to a lower energy at 531.717 eV, and the P–OH peak shifts to the lower energy at 532.481 eV with reduced intensity. The P=O peak in the H_2_PO_4_^–^ ion shows the double-bond character, and the P–OH peak shows the single-bond character.

**Figure 2:**
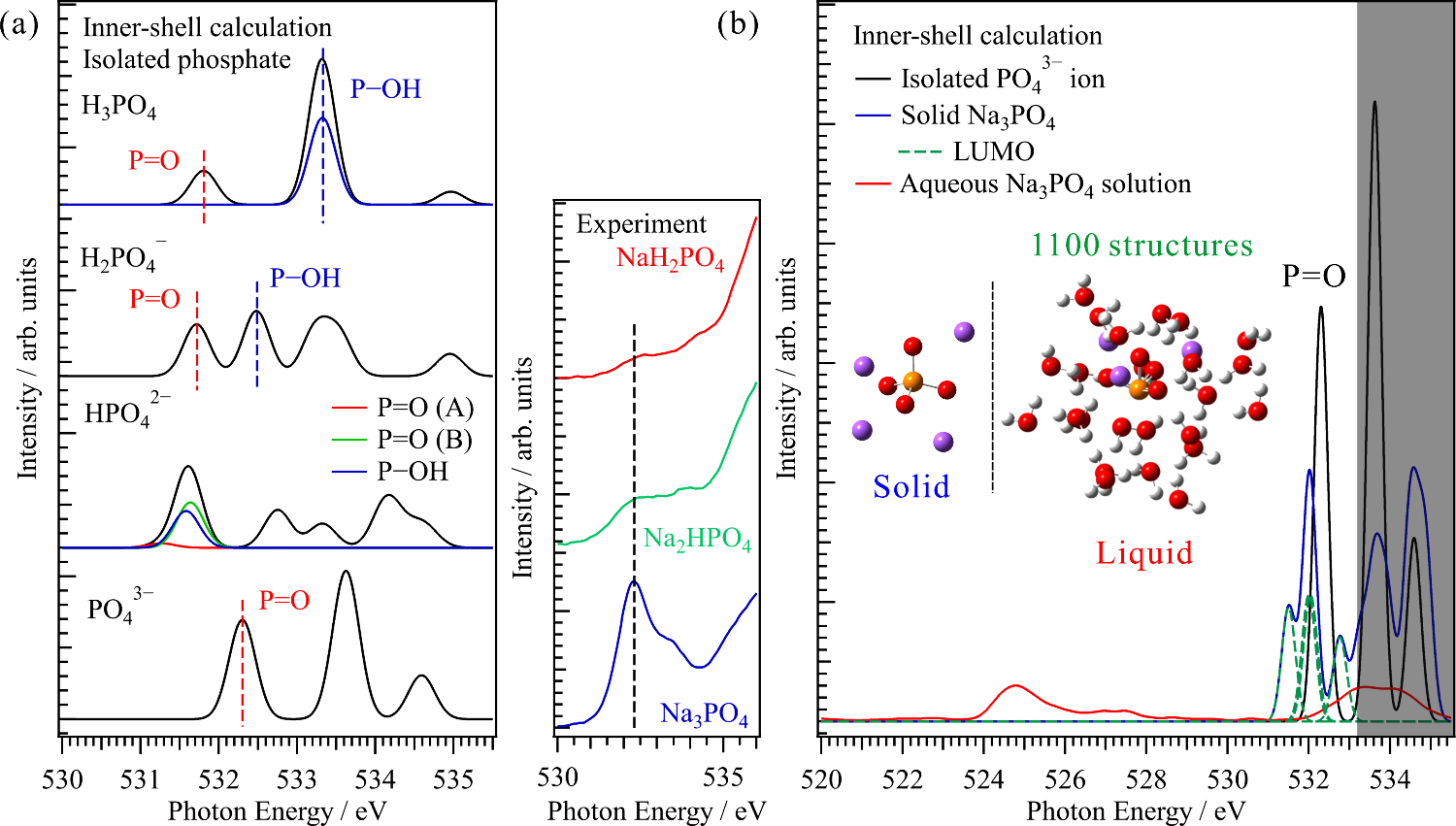
(a) O K-edge inner-shell spectra of isolated phosphate molecules. The dashed lines indicate the energetic positions of the P=O and P–OH peaks. Note that two P=O peaks (A and B) exist in the HPO_4_^2–^ ions. The experimental XAS spectra of solid phosphates are also shown for comparison. (b) O K-edge inner-shell spectra of isolated PO_4_^3–^ ions, solid Na_3_PO_4_, and aqueous Na_3_PO_4_ solution. The LUMO peaks of the P=O groups in solid Na_3_PO_4_ are also shown. The gray shaded energy regions show the higher inner-shell excited peaks, which were not considered in the present study. The inset shows the structures of solid Na_3_PO_4_. The inner-shell spectrum of aqueous Na_3_PO_4_ solution was calculated using 1100 molecular structures extracted from the snapshots of the molecular dynamics simulation, as shown in the inset.

In the HPO_4_^2–^ ion, the P=O peaks comprise two peaks: The P=O (A) peak at 531.267 eV is derived from one P=O group with a charge of – 1.301 and P–O bond length of 1.588 Å, and the P=O (B) peak at 531.636 eV is derived from two P=O groups with a charge of – 1.260 and P–O bond length of 1.572 Å. The P–OH peak is obtained at 531.582 eV, which is between two P=O peaks. The P–OH peak shows single-bond character. The P=O (B) peak shows double-bond character, which is larger than the P=O peak in the H_2_PO_4_^–^ ion. It is consistent with the increases of the P=O π* peaks in solid Na_2_HPO_4_ compared to those in solid NaH_2_PO_4_, as shown in Figure 1(a). The double-bond character of the P=O (A) peak is decreased compared to the P=O (B) peak because the P–O bond length of the P=O (A) group is longer than that of the P=O (B) group, which would be caused by the influence of the P–OH group.

In the PO_4_^3–^ ion, all the oxygen atoms are equivalent, exhibiting the highest negative charge of – 1.398 and longest P–O bond length of 1.637 Å compared with the P=O group of other phosphates. The P=O peak at 532.300 eV exhibits a high intensity, consistent with the pronounced P=O π* peaks in the XAS spectrum of solid Na_3_PO_4_, as shown in Figure 1(a). The double-bond character of the P=O peak in the PO_4_^3–^ ion is largest compared to that of other phosphates. Inner-shell calculations support the enhancement of the double-bond character in phosphate groups as the negative charge of the phosphates increases. The increase in the double-bond character of the phosphate groups is related to the reactivity of ATP hydrolysis, which increases above pH 8.^6^

Figure 2(b) shows the O K-edge inner-shell spectrum of solid Na_3_PO_4_ considering the previous crystallographic study,^45^ which was obtained from the Materials Project.^46^ The inner-shell spectra of solid NaH_2_PO_4_ and Na_2_HPO_4_ were shown in Sec. S7 of the Supporting Information. The molecular distances between phosphate ions and Na^+^ ions used in the structural models of solid phosphates were described in Sec. S8 of the Supporting Information. Although the P=O groups in the isolated PO_4_^3–^ ion consist of the single peak at 532.300 eV, the P=O peaks in the solid Na_3_PO_4_ are separated with three peaks, whose energetic positions are 531.515, 532.017, and 532.771 eV. These peak separations were caused by the interactions of different Na^+^ ions and would explain two P=O π* peaks at 532.3 and 533.4 eV in the O K-edge XAS spectrum of solid Na_3_PO_4_ shown in Figure 1(a). In the inner-shell spectrum of solid Na_2_HPO_4_, the P=O peaks show lower energy shifts and the P–OH peaks show higher energy shifts owing to the interactions of Na^+^ ions compared to that of isolated HPO_4_^2–^ ion. Although the P=O peaks in the isolated HPO_4_^2–^ ion show lower energetic positions than those in the isolated PO_4_^3–^ ion in the inner-shell spectra, the energetic positions of the P=O π* peaks between solid Na_2_HPO_4_ and Na_3_PO_4_ are opposite ones in the XAS spectra shown in Figure 1(a). This discrepancy would be solved by considering the interaction of Na^+^ ions because the P=O peaks show different lower energy shifts owing to the interactions of different Na^+^ ions. In the inner-shell spectrum of solid NaH_2_PO_4_, the P=O and P–OH peaks show larger energy shifts than those in the isolated H_2_PO_4_^–^ ions due to the interaction of Na^+^ ions. The weak P=O π* peaks in the XAS spectrum of solid NaH_2_PO_4_ would be caused by broad spectral shapes of the P=O π* peaks caused by the interactions of Na^+^ ions.

Figure 2(b) also shows the inner-shell spectrum of aqueous Na_3_PO_4_ solution calculated using the 1100 molecular structures extracted from the snapshots of the molecular dynamics simulation using GROMACS 2022.4.^47^ The molecular structures consist of PO_4_^3–^ ions surrounding by Na^+^ ions and water molecules within the molecular distance of 5.7 Å from the central PO_4_^3–^ ions, considering the radial distribution functions of PO_4_^3–^ ions described in Sec. S9 of the Supporting Information. The inner-shell spectrum includes the deviation of liquid structures owing to averaging the snapshots of the molecular dynamics simulation.^48^ The intensities of the P=O peaks in aqueous Na_3_PO_4_ solution were drastically decreased compared to those in solid Na_3_PO_4_ and isolated PO_4_^3–^ ions, which were consistent with the XAS spectra of aqueous phosphate solutions shown in Figure 1(b). Note that the P=O peaks in aqueous Na_3_PO_4_ solution show the lower energetic position at approximately 525 eV. The large discrepancy of the energetic positions between solid and liquid phases would be caused by strong structural deviation of phosphate ions by the interactions of Na^+^ ions and solvent water molecules. The molecular distances of Na^+^ ions with the PO_4_^3–^ ions in aqueous Na_3_PO_4_ solution were shorter than those in solid Na_3_PO_4_, as described in Sec. S9 of the Supporting Information. The solvent water molecules also approach the PO_4_^3–^ ions owing to the hydration structures of Na^+^ ions. The double-bond character of phosphate ions is decreased by the interaction of Na^+^ ions and the formation of hydration structures, resulting in the intensity decreases of the P=O π* peaks in aqueous solutions. The deviations in the molecular distances of the PO_4_^3–^ ions with Na^+^ ions and water molecules would cause the broad spectral shapes of the P=O π* peaks in the XAS spectrum of aqueous Na_3_PO_4_ solution.

Figure 3 shows the O K-edge XAS spectra of solid diphosphates with high purity: Na_2_H_2_P_2_O_7_ (>99.0%) and Na_4_P_2_O_7_ (>95%). The XAS spectrum of solid Na_2_H_2_P_2_O_7_ shows the shoulder peak at 534.3 eV, which would be derived from the P–OH bonds. Therefore, the XAS spectrum of Na_2_H_2_P_2_O_7_ does not show the P=O π* peak. The XAS spectrum of solid Na_4_P_2_O_7_ shows a broad profile in the energy region of the P=O π* peak and has a peak at 532.4 eV. This implies that the double-bond character of the diphosphates is also enhanced by increasing the negative charges of the phosphate groups. These results demonstrate that the change in the double-bond character of the phosphate groups is a general phenomenon applicable to high-energy phosphate compounds.

**Figure 3:**
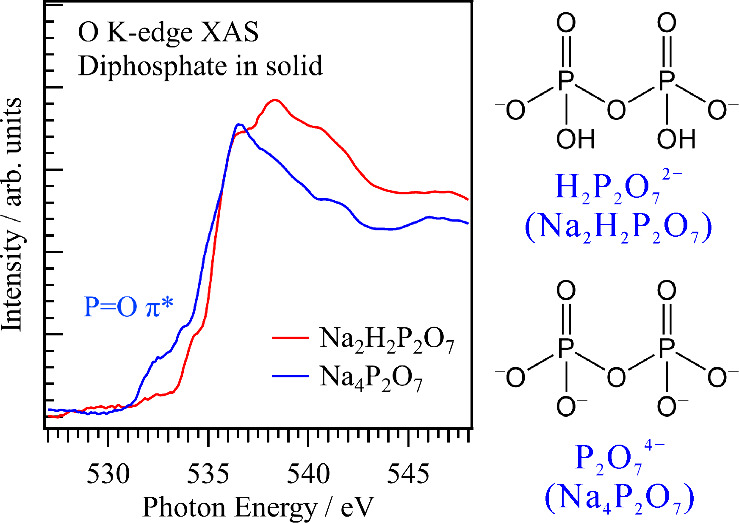
O K-edge XAS spectra of diphosphates in solid phases. The inset shows the molecular structures of diphosphates.

In conclusion, the double-bond character of the phosphates in solid and liquid phases was investigated based on the P=O π* peaks in O K-edge XAS spectra. The P=O π* peaks show small intensities in solid NaH_2_PO_4_ but were prominent for solid Na_2_HPO_4_ and Na_3_PO_4_. Inner-shell calculations validated that the double-bond character of the phosphate groups were enhanced by increasing the negative charges. The interactions of Na^+^ ions in solid phosphates cause the peak separations of the P=O π* peaks, resulting in the broad spectral shapes in the XAS spectra of solid phosphates. The XAS spectra of any phosphates in liquid phases exhibit no P=O π* peaks because the double-bond character of phosphate ions is decreased by the interactions of Na^+^ ions and the formation of hydration structures in aqueous solutions. The changes in the double-bond character of the phosphate groups constitute a general phenomenon, which were validated by the XAS measurements of solid diphosphates. The present study indicated that the double-bond character of phosphate groups is changed to chemical environments such as valences and interactions with surrounding molecules and can be probed from the electronic structures of the P=O π* orbitals in the O K-edge XAS. The reactivity of various biological phenomena such as ATP hydrolysis are related to changes in the double-bond character of the phosphate group under the influence of pH, the interactions of Na^+^ ions, and hydration.

## Supplementary Material



## Data Availability

The data underlying this study are openly available in Figshare at DOI: 10.6084/m9.figshare.29320325.^49^

## Notes

Experimental and theoretical methods; O K-edge XAS spectra of solid phosphates including water molecules; titration of phosphoric acid; O K-edge XAS spectrum of 1 M Na_3_PO_4_ aqueous solution; calculated charge distributions of isolated phosphate molecules; bond character of LUMO orbitals in isolated phosphate molecules; calculated inner-shell spectra of solid NaH_2_PO_4_ and Na_2_HPO_4_; molecular distances in structural models of solid phosphates; and radial distribution function of aqueous Na_3_PO_4_ solution (PDF)
