# Supplementary material for: Double-Bond Character of Phosphates in Solid and Liquid Phases Probed by Oxygen K‑Edge X‑ray Absorption Spectroscopy
Source: J Phys Chem Lett. 2026 Jun 1;17(23):6432–7. doi: 10.1021/acs.jpclett.6c00737 (PMC13267085; doi:10.1021/acs.jpclett.6c00737)
Supplement: Supplementary file 1 [file jz6c00737_si_001.pdf]

Supporting Information

Double-Bond Character of Phosphates in Solid and Liquid Phases Probed by Oxygen K-Edge X-ray Absorption Spectroscopy

Masanari Nagasaka,[*,1,2] Fumitoshi Kumaki,[3,4] and Jun-ichi Adachi [3,5]

[1] *Institute for Molecular Science, Okazaki 444-8585, Japan*

[2] *Molecular Science Program, Graduate Institute for Advanced Studies, SOKENDAI, Okazaki 444-8585, Japan*

[3] *Institute of Materials Structure Science, High Energy Accelerator Research Organization, Tsukuba, Ibaraki 305-0801, Japan*

[4] *Department of Chemistry, Keio University, Yokohama 223-8522, Japan*

[5] *Materials Structure Science Program, Graduate Institute for Advanced Studies, SOKENDAI, Tsukuba, Ibaraki 305-0801, Japan*

*Corresponding Author
E-mail: nagasaka@ims.ac.jp

Table of Contents **Page**

S1. Experimental and theoretical methods S2
S2. O K-edge XAS spectra of solid phosphates including water molecules S4
S3. Titration of phosphoric acid S5
S4. O K-edge XAS spectrum of 1 M Na_3PO_4 aqueous solution S5
S5. Calculated charge distributions of isolated phosphate molecules S6
S6. Bond character of LUMO orbitals in isolated phosphate molecules S7
S7. Calculated inner-shell spectra of solid NaH_2PO_4 and Na_2HPO_4 S7
S8. Molecular distances in structural models of solid phosphates S8
S9. Radial distribution function of aqueous Na_3PO_4 solution S9

References S10

S1. Experimental and theoretical methods

S1.1. Sample preparation

The high-purity solid phosphates and diphosphates were obtained from Sigma-Aldrich: NaH_2PO_4 (>99.0%), Na_2HPO_4 (>99.0%), Na_3PO_4 (>99.0%), $Na_2H_2P_2O_7$ (>99.0%), and $Na_4P_2O_7$ (>95%). $Na_3PO_4 \cdot 12H_2O$ (>99.0%) were obtained from Wako Pure Chemicals. Phosphoric acid was obtained from Wako Pure Chemicals. A 1 M NaOH solution was obtained from Nacalai Tesque. Ultrapure water with a resistivity above 18 MΩ was utilized.

Phosphate ions were prepared via pH titration, as shown in Sec. S3. The 200 mM phosphoric acid at pH of 1.53 was used to prepare the H_3PO_4 solution. The $H_2PO_4^-$ solution with pH of 5.38 was obtained at the first neutralization point of 200 mM H_3PO_4 solution by adding 1 M NaOH. The HPO_4^{2-} solution with pH of 9.99 was prepared at the second neutralization point of 200 mM H_3PO_4 solution by adding 1 M NaOH. The PO_4^{3-} solution with pH of 12.22 were prepared by mixing 10 ml of 200 mM H_3PO_4 solution with 5 ml of 1 M NaOH, which exceeds the third neutralization point. The concentration of the PO_4^{3-} solution was 133 mM.

S1.2. Soft X-ray absorption spectroscopy of solid samples

The O K-edge X-ray absorption spectroscopy (XAS) measurements of phosphates and diphosphates in solid phases were performed at the soft X-ray beamline BL3U at the UVSOR-III Synchrotron.[1] The energy resolution of the incident soft X-rays was 0.4 eV. Solid phosphate samples were pressed to indium plates onto a stainless sample holder, which was installed on a vacuum chamber in the ultrahigh vacuum condition below 10^{-5} Pa. The XAS spectra were obtained in total electron yield by measuring sample drain currents caused by soft X-ray absorption processes. The photon energies were precisely calibrated by measuring the O K-edge XAS spectra of the polymer film before and after the sample measurements.[2]

S1.3. Soft X-ray absorption spectroscopy of liquid samples

The O K-edge XAS measurements of phosphates in liquid phases were performed at the soft X-ray beamline BL-7A of the Photon Factory, Institute of Materials Structure Science, High Energy Accelerator Research Organization (KEK-PF).[3] The energy resolution of soft X-rays was 0.29 eV. The transmission-type liquid cell for XAS was placed at ambient pressure conditions of helium gas, where a liquid layer was sandwiched between two 100-nm-thick Si_3N_4 membranes.[4, 5] The thickness of the liquid layer was controlled by adjusting the helium pressure around the liquid cell. The beam size of soft X-rays was 200 × 200 μm^2 owing to the window size of the Si_3N_4 membrane, which separates the chamber of the liquid cell in the atmospheric helium condition and the soft X-ray beamline under an ultrahigh vacuum condition. The XAS spectra were obtained using the Beer-Lambert law, $\ln(I_0/I)$, where I_0 and I are the transmission signals of the bare Si_3N_4 membranes and liquid samples confined by the Si_3N_4 membranes, respectively. Liquid samples were exchanged using a syringe pump. The photon energies were calibrated precisely by measuring the XAS spectra of the polymer film before and after the sample measurements.[2]

S1.4. Structural optimization of solid phosphates

The structures of the isolated phosphate molecules were optimized with MP2/aug-cc-pVDZ using Gaussian 16.[6] The model structures of solid phosphates were constructed by adding Na^+ ions around the isolated phosphate ions considering the previous crystallographic studies obtained from the Materials Project from database version v2025.09.25.[7] The crystallographic structure of NaH_2PO_4 was retrieved from the database mp-24109.[8] The model structure of solid NaH_2PO_4 consists of one $H_2PO_4^-$ ion with Na^+ ions within the distance of 3.5 Å from the P atom of the $H_2PO_4^-$ ion. The crystallographic structure of Na_2HPO_4 was retrieved from the database mp-703305.[9] The model structure of solid Na_2HPO_4 consists of one HPO_4^{2-} ion with Na^+ ions within the distance of 3.5 Å from the P atom of the HPO_4^{2-} ion. The crystallographic structure of Na_3PO_4 was retrieved from the database mp-4223.[10] The model structure of solid Na_3PO_4 consists of one PO_4^{3-} ion with Na^+ ions within the distance of 3.2 Å from the P atom of the PO_4^{3-} ion.

S1.5. Inner-shell calculations of solid phosphates

The O K-edge inner-shell calculations were conducted using the program package GSCF3.[11, 12] The molecular structures of isolated phosphate molecules and solid phosphates were described in Sec. S1.4. The ground and core excited states were calculated through the Hartree-Fock method, namely, ΔSCF (self-consistent field). The core-hole was frozen on a specified O atom in the SCF calculations for the core excited state. The relaxed Hartree–Fock potential for the O 1s ionized state was obtained using a partial SCF calculation within the orbital manifold orthogonalized to the valence excited state. The present calculations do not include zero-point vibrational energy. The contracted Gaussian-type functions by Huzinaga *et al.* were used as primitive basis functions: (73/7) for C, N, and O, (533/53) for P, and (6) for H.[13] In the phosphate molecules, the contraction schemes were (3111121/3112/1*) for the O atoms, (3112121/31121/1*) for the P atoms, and (42) for H atoms. The *d*-type polarization functions were also used at the O atoms ($\zeta_d = 1.154$) and the P atoms ($\zeta_d = 0.340$). The contraction scheme in the Na^+ ion was (43/4). In the inner-shell calculations of aqueous Na_3PO_4 solutions shown in Sec. S1.7, the contraction schemes in the water molecules were (51121/52) for the O atoms and (42) for H atoms. The calculated inner-shell spectra were obtained by plotting the intensities and energies of several unoccupied orbitals excited from the oxygen atoms in the phosphate groups and were convoluted by Gaussian profiles with the width of 0.4 eV.

S1.6. Molecular dynamics simulation of aqueous Na_3PO_4 solution

Molecular dynamics (MD) simulation of aqueous Na_3PO_4 solution was performed using the program package GROMACS 2022.4.[14] The potential of the PO_4^{3-} ion was generated using ACPYPE server.[15, 16] The potential of the Na^+ ion was represented by the Amber03 model.[17] The potential of the water molecule was represented by the TIP4P/Ew model.[18] The cubic boxes of the MD simulations included one PO_4^{3-} ion, three Na^+ ions, and 416 water molecules for representing aqueous Na_3PO_4 solution with the concentration of 133 mM. The temperature was controlled using the Nosé-Hoover thermostat method.[19, 20] The pressure was adjusted using the Parrinello-Rahman method.[21] The simulations were performed at a time step of 1 fs with periodic boundary

conditions and the partial-mesh Ewald method.[22] The equilibrium structures were obtained by the simulations, which run during 500 ps at −173.15 °C in the canonical ensemble, 500 ps at −73.15 °C and 1 atm and 2 ns at 25 °C and 1 atm in the isobaric-isothermal ensemble. The equilibrium structures were sampled at a time step of 1 ps at 25 °C and 1 atm for a simulation time of 10 ns.

S1.7. Inner-shell calculations of aqueous Na_3PO_4 solution

The O K-edge inner-shell spectrum of aqueous Na_3PO_4 solution was obtained using 1100 molecular structures of PO_4^{3-} ions surrounded by Na^+ ions and solvent water molecules. These molecular structures were extracted from the snapshots of the MD simulation, as described in Sec. S1.6. Considering the radial distribution functions (RDF) of PO_4^{3-} ions with solvent water molecules and Na^+ ions shown in Sec. S9, the Na^+ ions were extracted within 5.7 Å of the P atoms, and the solvent water molecules were extracted when the molecular distances between the O atoms of water molecules and the P atoms were within 5.7 Å. The inner-shell spectra of PO_4^{3-} ions with Na^+ ions and solvent water molecules were calculated using the program package GSCF3,[11, 12] as described in Sec. S1.5. The inner-shell spectrum of PO_4^{3-} ions in aqueous solution was obtained by averaging 1100 inner-shell spectra of the extracted molecular structures during the 10 ns production run of the MD simulations. Because the molecular structures from the snapshots of the MD simulation include the deviation of liquid structures,[23] the calculated inner-shell spectrum includes the structural deviations of the PO_4^{3-} ions caused by the interactions of Na^+ ions and water molecules.

S2. O K-edge XAS spectra of solid phosphates including water molecules

Figure S1 shows O K-edge XAS spectra of solid Na_3PO_4 and $Na_3PO_4 \cdot 12H_2O$. The P=O π^* peaks in the solid $Na_3PO_4 \cdot 12H_2O$ consist of two peaks at 532.4 eV and 533.4 eV. The P=O π^* peak intensity of solid $Na_3PO_4 \cdot 12H_2O$ is decreased owing to the interactions of water molecules compared to those of solid Na_3PO_4.

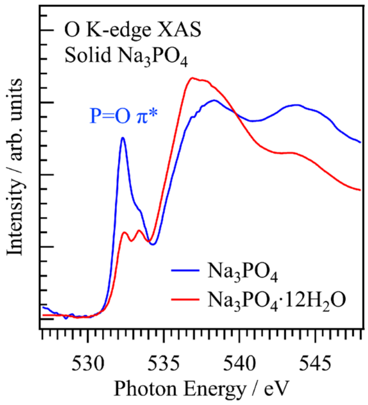

Fig. S1. O K-edge XAS spectra of solid Na_3PO_4 and $Na_3PO_4 \cdot 12H_2O$.

S3. Titration of phosphoric acid

Figure S2 shows the change in pH during the titration for 10 ml of 200 mM H_3PO_4 solution with 1 M NaOH solution. Titration measurements were performed employing a pH meter (Mettler Toledo, FiveEasy FP20). The H_3PO_4 solution was obtained without the addition of 1 M NaOH. The $H_2PO_4^-$ solution was obtained at the first neutralization point near pH = 4. The HPO_4^{2-} solution was obtained at the second neutralization point near pH = 9. The PO_4^{3-} solution was obtained exceeding the third neutralization point near pH = 12.

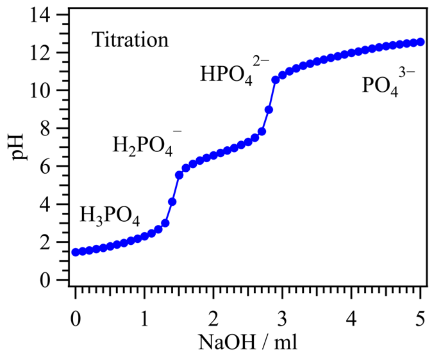

Fig. S2. Titration for 10 ml of 200 mM H_3PO_4 aqueous solution with 1 M NaOH solution.

S4. O K-edge XAS spectrum of 1 M Na_3PO_4 aqueous solution

Figure S3 shows O K-edge XAS spectrum of 1 M Na_3PO_4 aqueous solution at 25 °C, prepared by dissolving solid $Na_3PO_4 \cdot 12H_2O$ with ultrapure water. The strong absorbance at approximately 535 eV originates from solvent water. In contrast to the XAS spectrum of solid Na_3PO_4, the P=O π^* peak of the PO_4^{3-} ions was not observed in aqueous solution despite the high concentration of 1 M Na_3PO_4 aqueous solution, which is sufficiently above the detection threshold (100 mM) of the O K-edge XAS measurement.[24]

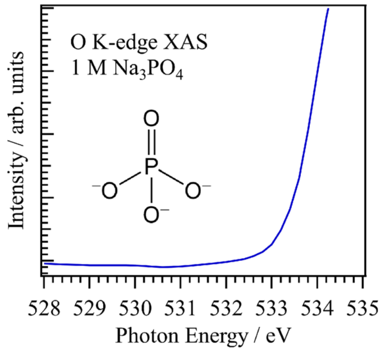

Fig. S3. O K-edge XAS spectrum of 1 M Na_3PO_4 aqueous solution.

S5. Calculated charge distributions of isolated phosphate molecules

Figure S4 shows the optimized molecular structures of isolated phosphate molecules. The charge distributions were calculated using Gaussian 16.[6] Table S1 lists the charges on the oxygen atoms and the corresponding P–O bond lengths for each species.

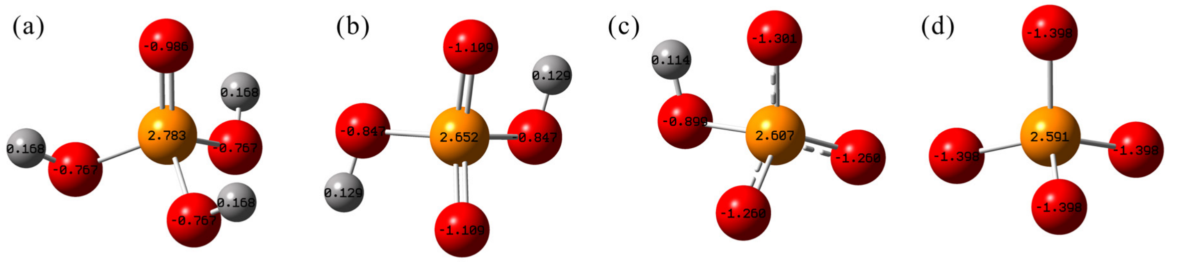

Fig. S4. Optimized molecular structures of isolated phosphate molecules with charge distributions. (a) H_3PO_4, (b) $H_2PO_4^-$, (c) HPO_4^{2-}, and (d) PO_4^{3-}.

Table S1. Calculated charges on the oxygen atoms and the corresponding P–O bond lengths of the phosphate groups.

Molecule	Functional group	Charge on oxygen atom	P–O bond length / Å
H_3PO_4	P=O	−0.986	1.505
	P–OH	−0.767	1.634
$H_2PO_4^-$	P=O	−1.109	1.533
	P–OH	−0.847	1.704
HPO_4^{2-}	P=O (A)	−1.301	1.588
	P=O (B)	−1.260	1.572
	P–OH	−0.899	1.806
PO_4^{3-}	P=O	−1.398	1.637

The H_3PO_4 molecule contains both P=O and P–OH groups. The P=O group exhibits a higher negative charge and shorter P–O bond length than the P–OH group. Compared with the H_3PO_4 molecule, the $H_2PO_4^-$ ion exhibits more negative charge and longer P–O bond length of the P=O group. The P–OH group also exhibits more negative charge and longer P–O bond length. This tendency continues in the HPO_4^{2-} ion, wherein both the P=O and P–OH groups exhibit more negative charges and longer P–O bond lengths. The P=O groups can be categorized into two different types: One P=O (A) group exists with a negative charge of −1.301 and P–O bond length of 1.588 Å, and two P=O (B) groups exist with negative charges of −1.260 and P–O bond lengths of 1.572 Å. P=O (A) group exhibits more negative charge and longer P–O bond length compared with that of P=O (B) group. In the PO_4^{3-} ion, all the P=O groups become equivalent, each exhibiting a negative charge of −1.398 and P–O bond length of 1.637 Å. These are the highest negative charges and longest P–O bond lengths among the phosphates examined.

S6. Bond character of LUMO orbitals in isolated phosphate molecules

Table S2 shows the bond character of LUMO orbitals in isolated phosphate molecules, which were calculated using the program package GSCF3.[11, 12] The *s*-type orbital represents single-bond character, and the *p*-type orbital represents double-bond character. Note that the total summations of *s*-type and *p*-type orbitals are a little bit deviated from 100%.

Table S2. The bond character of LUMO orbitals in isolated phosphate molecules. The *s*-type orbital represents single-bond character, and the *p*-type orbital represents double-bond character.

Molecule	Functional group	*s*-type orbital (%)	*p*-type orbital (%)
H_3PO_4	P=O	68.9	28.6
	P–OH	93.6	6.4
	P–OH	93.9	5.9
	P–OH	93.7	6.2
$H_2PO_4^-$	P=O	72.1	25.6
	P=O	72.1	25.7
	P–OH	101.2	−1.4
	P–OH	101.2	−1.4
HPO_4^{2-}	P=O (A)	85.5	14.4
	P=O (B)	65.7	31.2
	P=O (B)	65.7	31.2
	P–OH	103.2	−3.5
PO_4^{3-}	P=O	57.3	42.8
	P=O	57.3	42.9
	P=O	57.3	42.9
	P=O	57.3	42.9

S7. Calculated inner-shell spectra of solid NaH_2PO_4 and Na_2HPO_4

Figure S5(a) shows the O K-edge inner-shell spectrum of solid NaH_2PO_4 considering the previous crystallographic study,[8] together with that of isolated $H_2PO_4^-$ ions. In the isolated $H_2PO_4^-$ ions, the P=O peak shows the energetic position of 531.717 eV and the P–OH peak shows that of 532.481 eV. In the solid NaH_2PO_4, the P=O peaks are separated by the interaction of Na^+ ions, where the P=O peak at the lower energy side shows the energetic position of 530.230 eV. The P–OH peaks are also separated by the interaction of Na^+ ions. Because the P=O and P–OH peaks in solid NaH_2PO_4 show larger energy shifts than those in the $H_2PO_4^-$ ions by the interaction of Na^+ ions, the XAS spectrum of solid NaH_2PO_4 would show the broad spectral shapes of the P=O π^* peaks.

Figure S5(b) shows the O K-edge inner-shell spectrum of solid Na_2HPO_4 considering the previous

crystallographic study,[9] together with that of isolated HPO_4^{2-} ions. The energetic positions of the P=O and P–OH peaks are close to each other in the isolated HPO_4^{2-} ions. In the solid Na_2HPO_4, the P=O peak shows a lower energy shift, whose energetic position was 531.319 eV, and the P–OH peak shows a higher energy shift owing to the interactions of Na^+ ions compared to that of isolated HPO_4^{2-} ions.

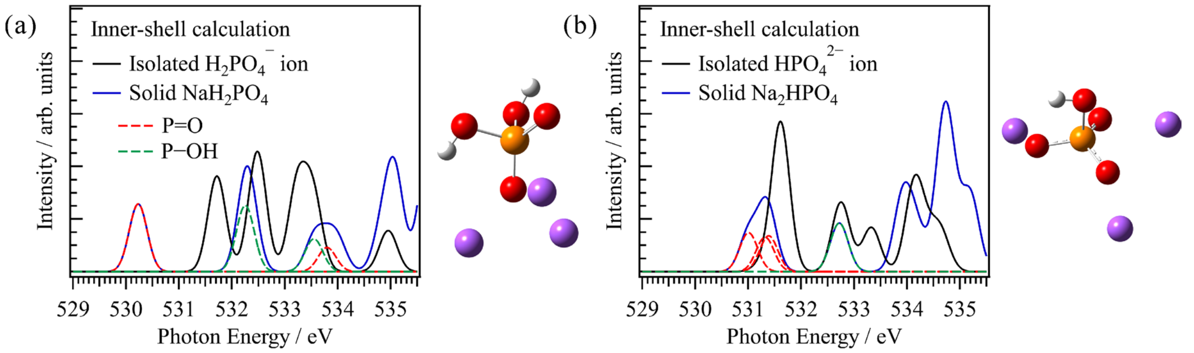

Fig. S5. (a) The O K-edge inner-shell spectra of isolated $H_2PO_4^-$ ions and solid NaH_2PO_4. The inset shows the structures of solid NaH_2PO_4. (b) The O K-edge inner-shell spectra of isolated HPO_4^{2-} ions and solid Na_2HPO_4. The inset shows the structures of solid Na_2HPO_4. The dashed lines are derived from the spectral contributions from the LUMO orbitals in the P=O and P–OH groups in solid NaH_2PO_4 and Na_2HPO_4.

S8. Molecular distances in structural models of solid phosphates

Table S3 shows the molecular distances between the P atoms of phosphate ions and Na^+ ions in solid phosphates, which were determined by the previous crystallographic studies retrieved from the Materials Project.[7]

Table S3. The molecular distances between the P atoms of phosphate ions and Na^+ ions in solid phosphates.

Solid phosphates	P – Na^+ / Å
NaH_2PO_4	2.977
	3.434
	3.477
Na_2HPO_4	3.010
	3.155
	3.433
Na_3PO_4	2.892
	3.002
	3.115
	3.137

S9. Radial distribution function of aqueous Na_3PO_4 solution

Figure S6(a) shows RDF of the P atoms in the PO_4^{3-} ions with the oxygen atoms (O_w) of H_2O in aqueous Na_3PO_4 solution. The P–O_w peaks show the positions of 3.41 Å and 3.80 Å. Considering the second coordination shells of water molecules around the PO_4^{3-} ions, the inner-shell calculation of aqueous Na_3PO_4 solution used the model structures of PO_4^{3-} ions surrounded by Na^+ ions and solvent water molecules within the distances of 5.7 Å. Figure S6(b) shows RDF of the P atoms in the PO_4^{3-} ions with Na^+ ions. The P–Na^+ peak shows the position of 2.83 Å. The P–Na^+ distances in aqueous Na_3PO_4 solutions are shorter than those in solid Na_3PO_4, which were described in Sec. S8. Therefore, the interactions of Na^+ ions with the PO_4^{3-} ions in aqueous solution are stronger than those in solid phase, which would affect the inner-shell spectrum of aqueous Na_3PO_4 solution.

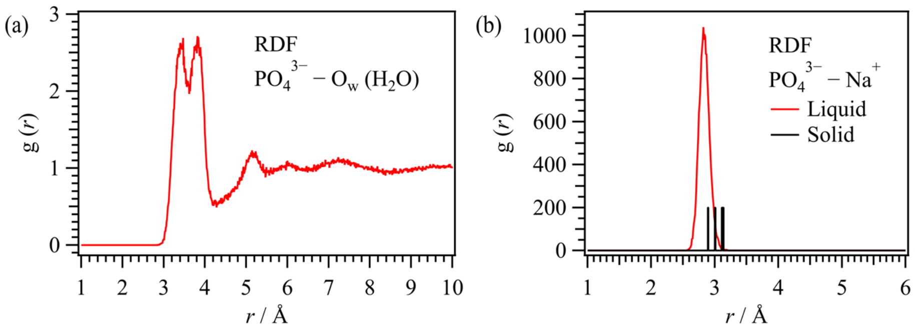

Fig. S6. (a) RDF of the P atoms in the PO_4^{3-} ions with O_w of H_2O in aqueous Na_3PO_4 solution. (b) RDF of the P atoms in the PO_4^{3-} ions with Na^+ ions in aqueous Na_3PO_4 solution. The molecular distances between the PO_4^{3-} ions and Na^+ ions in the model structures of solid Na_3PO_4 are also shown.

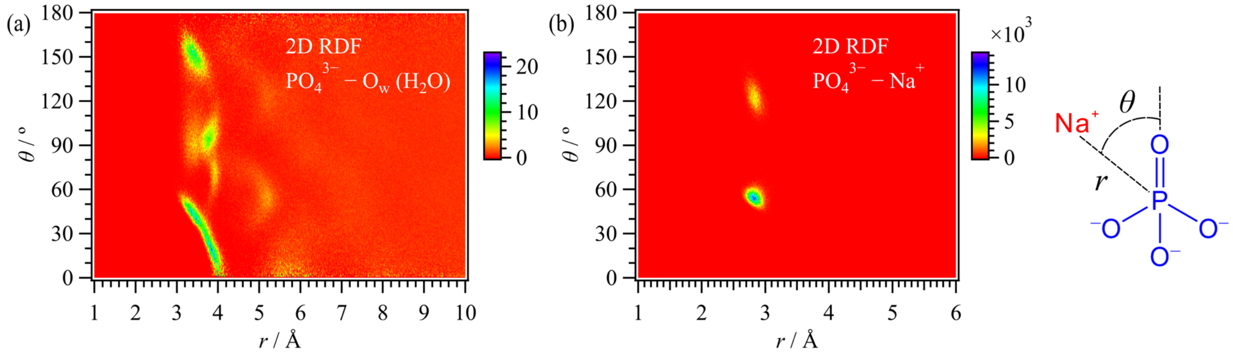

Fig. S7. (a) 2D RDF of the P atoms in the PO_4^{3-} ions with O_w of H_2O in aqueous Na_3PO_4 solution. (b) 2D RDF of the P atoms in the PO_4^{3-} ions with Na^+ ions in aqueous Na_3PO_4 solution. The horizontal axes are the distance r of the P atoms with the O_w atoms or Na^+ ions. The vertical axes are the angles θ, whose definitions are described in the inset.

Figure S7 shows the two-dimensional (2D) RDF (a) P–O_w and (b) P–Na^+ in aqueous Na_3PO_4 solution. The horizontal axes of both panels are the distance *r* between two atoms. The vertical axes of both panels are the angle θ between the P=O bond of the PO_4^{3-} ion and *r*. The angle θ in the 2D RDF of P–Na^+ is 54°, indicating that Na^+ ions exist between two P=O groups in the PO_4^{3-} ion. In the 2D RDF of P–O_w, the molecular distances of water molecules from the PO_4^{3-} ions are shortest at θ = 54°, which are close to those of Na^+ ions. It means that solvent water molecules approach the PO_4^{3-} ions owing to the hydration structures of Na^+ ions.

References

(1) Hatsui, T.; Shigemasa, E.; Kosugi, N. Design of a Transmission Grating Spectrometer and an Undulator Beamline for Soft X-ray Emission Studies. *AIP Conf. Proc.* **2004,** *705*, 921-924.

(2) Nagasaka, M.; Yuzawa, H.; Horigome, T.; Kosugi, N. Reliable Absorbance Measurement of Liquid Samples in Soft X-ray Absorption Spectroscopy in Transmission Mode. *J. Electron Spectrosc. Relat. Phenom.* **2018,** *224*, 93-99.

(3) Amemiya, K.; Kondoh, H.; Yokoyama, T.; Ohta, T. A Soft X-ray Beamline for Surface Chemistry at the Photon Factory. *J. Electron Spectrosc. Relat. Phenom.* **2002,** *124*, 151-164.

(4) Nagasaka, M.; Yuzawa, H.; Kosugi, N. Soft X-ray Absorption Spectroscopy of Liquids for Understanding Chemical Processes in Solution. *Anal. Sci.* **2020,** *36*, 95-105.

(5) Nagasaka, M.; Kosugi, N. Soft X-ray Absorption Spectroscopy for Observing Element-Specific Intermolecular Interaction in Solution Chemistry. *Chem. Lett.* **2021,** *50*, 956-964.

(6) Frisch, M. J.; Trucks, G. W.; Schlegel, H. B.; Scuseria, G. E.; Robb, M. A.; Cheeseman, J. R.; Scalmani, G.; Barone, V.; Petersson, G. A.; Nakatsuji, H.; et al. *Gaussian 16*; Wallingford, CT, 2019.

(7) Jain, A.; Ong, S. P.; Hautier, G.; Chen, W.; Richards, W. D.; Dacek, S.; Cholia, S.; Gunter, D.; Skinner, D.; Ceder, G.; et al. Commentary: The Materials Project: A Materials Genome Approach to Accelerating Materials Innovation. *APL Mater.* **2013,** *1*, 011002.

(8) Choudhary, R. N. P.; Nelmes, R. J.; Rouse, K. D. A Room-Temperature Neutron-Diffraction Study of NaH_2PO_4. *Chem. Phys. Lett.* **1981,** *78*, 102-105.

(9) Baldus, M.; Meier, B. H.; Ernst, R. R.; Kentgens, A. P. M.; zu Altenschildesche, H. M.; Nesper, R. Structure Investigation on Anhydrous Disodium Hydrogen Phosphate Using Solid-State NMR and X-ray Techniques. *J. Am. Chem. Soc.* **1995,** *117*, 5141-5147.

(10) Harrison, R. J.; Putnis, A.; Kockelmann, W. Phase Transition Behaviour and Equilibrium Phase Relations in the Fast-Ion Conductor System Na_3PO_4–Na_2SO_4. *Phys. Chem. Chem. Phys.* **2002,** *4*, 3252-3259.

(11) Kosugi, N.; Kuroda, H. Efficient Methods for Solving the Open-Shell SCF Problem and for Obtaining an Initial Guess - the One-Hamiltonian and the Partial SCF Methods. *Chem. Phys. Lett.* **1980,** *74*, 490-493.

(12) Kosugi, N. Strategies to Vectorize Conventional SCF-CI Algorithms. *Theor. Chim. Acta* **1987,** *72*, 149-173.

(13) Huzinaga, S.; Andzelm, J.; Klobukowski, M.; Radzio-Andzelm, E.; Sakai, Y.; Tatewaki, H., *Gaussian*

Basis Sets for Molecular Calculations, Elsevier: Amsterdam, 1984.

(14) Abraham, M. J.; Murtola, T.; Schulz, R.; Páll, S.; Smith, J. C.; Hess, B.; Lindahl, E. GROMACS: High Performance Molecular Simulations through Multi-Level Parallelism from Laptops to Supercomputers. *SoftwareX* **2015,** *1-2*, 19-25.

(15) Sousa da Silva, A. W.; Vranken, W. F. ACPYPE - AnteChamber PYthon Parser interfacE. *BMC Res. Notes* **2012,** *5*, 367.

(16) Kagami, L.; Wilter, A.; Diaz, A.; Vranken, W. The ACPYPE Web Server for Small-Molecule MD Topology Generation. *Bioinformatics* **2023,** *39*, btad350.

(17) Sorin, E. J.; Pande, V. S. Exploring the Helix-Coil Transition Via All-Atom Equilibrium Ensemble Simulations. *Biophys. J.* **2005,** *88*, 2472-2493.

(18) Horn, H. W.; Swope, W. C.; Pitera, J. W.; Madura, J. D.; Dick, T. J.; Hura, G. L.; Head-Gordon, T. Development of an Improved Four-Site Water Model for Biomolecular Simulations: TIP4P-Ew. *J. Chem. Phys.* **2004,** *120*, 9665-9678.

(19) Nosé, S. A Unified Formulation of the Constant Temperature Molecular Dynamics Methods. *J. Chem. Phys.* **1984,** *81*, 511-519.

(20) Hoover, W. G. Canonical Dynamics: Equilibrium Phase-Space Distributions. *Phys. Rev. A* **1985,** *31*, 1695-1697.

(21) Parrinello, M.; Rahman, A. Polymorphic Transitions in Single Crystals: A New Molecular Dynamics Method. *J. Appl. Phys.* **1981,** *52*, 7182-7190.

(22) Darden, T.; York, D.; Pedersen, L. Particle Mesh Ewald: An $N\cdot\log(N)$ Method for Ewald Sums in Large Systems. *J. Chem. Phys.* **1993,** *98*, 10089-10092.

(23) Nagasaka, M. Carbon K-Edge X-ray Absorption Spectra of Liquid Alcohols from Quantum Chemical Calculations of Liquid Structures Obtained by Molecular Dynamics Simulations. *J. Chem. Phys.* **2023,** *158*, 024501.

(24) Nagasaka, M.; Kumaki, F.; Yao, Y.; Adachi, J.; Mochizuki, K. Mechanism of Poly(*N*-isopropylacrylamide) Cononsolvency in Aqueous Methanol Solutions Explored *Via* Oxygen K-Edge X-ray Absorption Spectroscopy. *Phys. Chem. Chem. Phys.* **2024,** *26*, 13634-13638.
